# Gaseous Ozonation to Reduce Aflatoxins Levels and Microbial Contamination in Corn Grits

**DOI:** 10.3390/microorganisms7080220

**Published:** 2019-07-28

**Authors:** Yuri Duarte Porto, Felipe Machado Trombete, Otniel Freitas-Silva, Izabela Miranda de Castro, Gloria Maria Direito, José Luis Ramirez Ascheri

**Affiliations:** 1 Graduate Program in Food Science and Technology, Institute of Technology, Federal Rural University of Rio de Janeiro (UFRRJ), Seropédica 23897-000, Brazil; 2 Food Engineering Department, Federal University of São João del-Rei, Campus Sete Lagoas, Sete Lagoas 35701-970, Brazil; 3 Brazilian Agricultural Research Corporation, Embrapa Agroindústria de Alimentos, Rio de Janeiro 23020-470, Brazil; 4 Department of Microbiology and Immunology Veterinary, Institute of Veterinary, Federal Rural University of Rio de Janeiro (UFRRJ), Seropédica 23897-000, Brazil

**Keywords:** aflatoxins, decontamination, ozone

## Abstract

Corn is one of the most cultivated cereals in Brazil. However, its grains are constantly exposed to contamination by mycotoxins. Corn grits are used by the food industry to produce a large variety of corn products such as canjiquinha, a cultural food easily purchased by the Brazilian consumer at low prices. Some studies have demonstrated high contamination of this product by aflatoxins (AFs), representing a potential risk of exposure due to such a contamination. In this study, the efficacy of gaseous ozonation was evaluated on the levels of aflatoxins and on the microbial contamination of corn grits. The application of gaseous ozone was tested in different combinations of exposure time, ozone concentration, and canjiquinha mass. After the ozonation treatment, samples were collected for aflatoxin and microbiological analyses. Aflatoxins were evaluated using a high-performance liquid chromatography with fluorescence detection (HPLC-FD) system using pre-column derivatization, and the microbiological analyses were carried out for toxin-producer fungi and mesophilic bacteria. After ozone detoxification, results showed reductions up to 57% in aflatoxin levels. Total fungal count was reduced around 3.0 cycles log CFU g^−1^ and total mesophilic counts were reduced to non-detectable levels. These results demonstrated that ozonation is an effective alternative for reducing aflatoxin and microbial contamination in products like canjiquinha, thereby improving food safety.

## 1. Introduction

Maize or corn (*Zea mays* L.) is a cereal of extreme importance in the diets of many populations, contributing especially as a source of energy because of its high content of starches (up to 73% of kernel weight), proteins (8–13.7%), fatty acids (4–5.4%), as well as vitamins and minerals [1,2]. Corn grits are produced by removing the outer bran, the germ, and the tip cap of the grains, followed by grinding into smaller coarse bits. A wide variety of products can be produced from corn, depending on the size of the grain, such as soups, roasts, sweets, extruded products, and many varieties of corn snacks [3,4]. In Brazil, the product of ground corn kernels known as canjiquinha is widely consumed by the population and distributed due to its low cost. Some species of toxigenic fungi, such as *Aspergillus* spp., can develop in corn kernels and produce aflatoxins (AFs) under suitable conditions. These metabolites are highly toxic to humans and animals, especially the forms AFB1, AFB2, AFG1, and AFG2, which have been classified by the International Agency for Research on Cancer as genotoxic and carcinogenic molecules [5]. Different authors have reported aflatoxin contamination [6,7,8,9], demonstrating that it is a matter of public health concern, especially because corn grit products are consumed virtually throughout the world. The adoption of quality management systems throughout the corn production chain, such as good agricultural practices (GAPs), good manufacturing practices (GMPs), and hazard analysis and critical control points (HACCPs), is essential to ensure the safety of the corn grits in terms of contamination by mycotoxins [10,11]. On the other hand, when mycotoxins are already present in the kernels, some emerging technologies have been studied to reduce this contamination to safe levels [12]. The use of ozone (O_3_) has been considered an interesting method for the remediation of cereals contaminated by mycotoxins. The United States Food and Drug Administration (FDA) has recognized the ozone application as an oxidizing agent in food processing and as a generally recognized as safe (GRAS) substance for use [13]. When directly applied on cereal grains, the molecular O_3_ and the hydroxyl radicals (˙OH) generated in the process can react with mycotoxins, promoting their degradation to lower molecular weight products, thus eliminating or reducing their biological activity in terms of toxicity [14,15]. The efficacy of O_3_ in decontaminating mycotoxins depends on several factors, such as the O_3_ concentration, exposure time, moisture content, and temperature of the food. Using different conditions, some recent studies have demonstrated it is possible to obtain a high reduction in aflatoxins levels and microbiological contamination in cereal products [16,17]. However, more studies must be conducted to know the potential of O_3_ to reduce mycotoxins and microorganisms in a wider variety of cereal products, since the contamination of these foods is a relevant problem in terms of health and economics. In this study, we evaluated the effects of gaseous ozonation applied to corn grits, including the levels of aflatoxins (B1, B2, G1, and G2), fungal contamination, and total mesophilic count.

## 2. Materials and Methods

### 2.1. Chemicals and Reagents

Standard aflatoxins (AFB1, AFB2, AFG1, and AFG2) were purchased from Sigma-Aldrich (St. Louis, MO, USA). The reagents KCl and CuSO_4_ were ACS grade from Vetec (Rio de Janeiro, Rio de Janeiro, Brazil). Trifluoroacetic acid for HPLC was purchased from Tedia (São Paulo, São Paulo, Brazil). The organic solvents hexane, chloroform, acetonitrile, and methanol HPLC grade were acquired from Tedia (São Paulo, São Paulo, Brazil); ultrapure water was obtained using a Rios/Milli-Q^®^ purification system (Millipore, Danvers, MA, USA). Qualitative filter papers grade 1 were purchased from Whatman (Maidstone, Kant, UK), and the HPLC 13 mm polypropylene filters with 0.45 µm PVDF membrane Durapore^®^ (Millipore, Danvers, MA, USA) were purchased from Merck (São Paulo, São Paulo, Brazil). Plate count agar (PCA), used for total mesophilic count, Dichloran Rose Bengal Chloramphenicol (DRBC) agar, malt extract agar (MEA), and potato dextrose agar (PDA) used in fungal analyses were obtained from Himedia (Curitiba, Paraná, Brazil).

### 2.2. Sampling

The corn grit commercial product called canjiquinha used in this study was purchased from a cereal-processing factory located in Rio de Janeiro, Brazil. These corn grits were homogenized and stored in plastic barrels at 25 °C. All aliquots (25 g each) for aflatoxins and conidia artificial contamination used in this work were taken from this batch of samples. The aliquots were packed into 100% polyamide organza sacks and kept at −20 °C until the ozonation process.

#### Artificial Contamination of Samples

Aflatoxins. Following the experimental design, 25 g samples of homogenized corn grits were spiked with four aliquots of aflatoxin working solutions to obtain a final concentration of 50 µg kg^−1^ of each AF. Concentrations of the stock solutions were confirmed using UV spectrophotometer absorbance (Shimadzu UV-1201, Kyoto, Japan), according to the Association of Official Analytical Chemists (AOAC) (2005). Spiked samples were then packed into 100% polyamide organza fabric and kept at −3 °C until running the ozonation experiments.

Fungal samples. Here, 25 g samples of homogenized corn grits were fortified with a suspension of *Fusarium* spp. and *Aspergillus* spp. conidia with a count of 1.0 × 10^5^ conidia g^−1^.

Mesophilic bacteria samples. A homogenized corn aliquot of 25 g was extracted from the total mass of grains after ozonation (Table 1) in order to evaluate the natural population of mesophilic bacteria.

Control sample. A 25 g packet of corn grit sample was subjected to the ozonation process, where oxygen was used instead of ozone. This procedure represented the control of decontamination. A specific control was produced for each of the three sample types—aflatoxins, fungal samples, and mesophilic count. All assays concerning sample preparation for fungi and total mesophilic measurement were conducted inside a laminar flow cabinet (Veco, Campinas, São Paulo, Brazil).

### 2.3. Obtaining the Conidia Solution

*Fusarium* spp. and *Aspergillus* spp. were isolated from the corn grits in MEA and DRBC agar, with confirmation to genus level using an optical microscope (Olympus, BX51, Melville, LA, USA) [18]. Typical colonies were then inoculated separately into tubes containing PDA medium and incubated at 25 °C for five days for biomass production. To obtain the suspension of conidia of each fungus, 3 mL of 0.01% Tween in sterilized water were added to the tubes and mixed. The concentration of conidia mL^−1^ was calculated using a Neubauer chamber (Kasvi, São José dos Pinhais, Paraná, Brazil). Sufficient aliquots were taken from this suspension to fortify samples of corn grits to obtain a final count of approximately 1.0 × 10^5^ conidia g^−1^.

### 2.4. Ozonation System and Process

#### 2.4.1. Ozone Production

Ozone was obtained using an industrial ozonator (O & L 3.0 RM, Ozone & Life^®^, São José dos Campos, São Paulo, Brazil) supplied with 99.99% pure oxygen and a flow rate set at 0.5 L min^−1^. This device permits the O_3_ concentration control according to ten different corona discharge intensities; however, in this study the control of O_3_ concentration was determined by iodometric titration test from the output of the ozone generator [19].

#### 2.4.2. Ozonation System and Experimental Design

**Figure 1:**
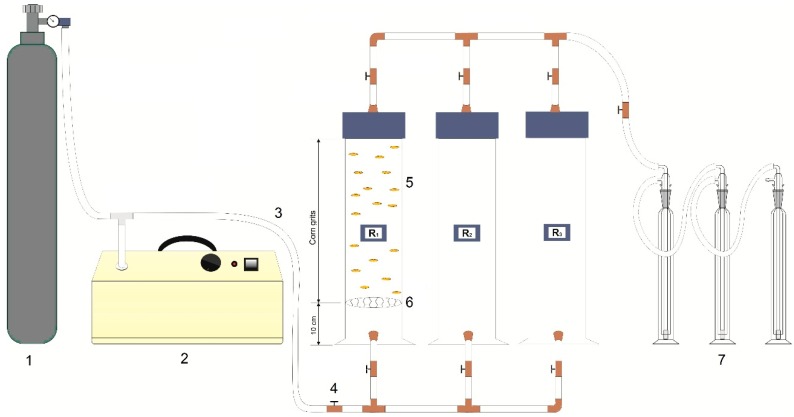
Ozonation system. 1—O_2_ cylinder/flux control valve; 2—Ozone generator; 3—Silicone hoses; 4—Copper connections; 5—PVC cylinders containing the corn grits; 6—Polyamide silicone support disk; 7—Gas wash bottles with potassium iodide solution.

The ozonation system was built using three experimental PVC cylinders (60 × 15 cm, length × diameter) filled with the corn grits, as shown in Figure 1. The grits were placed 10 cm above the base, supported by a disc made of polyamide fabric and silicon. The sample packets (25 g) for ozonation were placed on the top of the corn grits, and another 500 g were added to completely cover the packets. The amounts of grains used in each treatment are presented in the matrix design (Table 1). The ozonation of the corn grits was carried out using a full 2^3^ factorial design with combinations of three independent variables, namely, ozone concentration (20 to 60 mg/L), exposure time (120 to 480 min), and mass of grains (1 to 5 kg). The total aflatoxin level and the total fungal count were selected as dependent variables. These conditions were selected based on studies that investigated the effects of O_3_ on cereal grain quality. For each O_3_ condition, two replicates were performed. During each replicate, only one cylinder was used, while the other two remained closed. For the control samples, only O_2_ was passed through the grains at the same flow rate as under the experimental conditions (0.5 L min^−1^).

### 2.5. Analysis of Aflatoxins by HPLC

To evaluate the aflatoxin levels, the corn grit samples from the packets (25 g) used in the ozonation System were analyzed based on the method described previously [20]. First, 25 g of corn grits were transferred to an Erlenmeyer flask and 67.5 mL of methanol and 7.5 mL of 4% KCl solution were added and agitated for 60 min. Then, 75 mL of 10% CuSO_4_ and 7.5 g of celite were added to the flask. After precipitation, the extract was filtered through filter paper and 75 mL was collected and transferred to a separatory funnel with 75 mL of water. This solution was defatted by partition using 2 × 25 mL of hexane. Subsequently, 2 × 25 mL of chloroform were added to the separatory funnel, with vigorous shaking for 60 s. The chloroform phase containing the aflatoxins was collected and evaporated in a water bath until dryness. Aflatoxins were derived with water, trifluoroacetic acid, and acetonitrile (ratio of 7:2:1, v/v/v) according to the AOAC [21]. The quantification was carried out in an HPLC system, using a fluorescence detector (Agilent 1100 Series, Waldbronn, Germany) at 365 nm excitation and 450 nm emission, a Rheodyne injector (20 µL), a reverse phase C18 column (Ace, 250 mm × 4.6 mm, 5 µm) and a mobile phase, consisting of water, methanol, and acetonitrile (ratio of 7:2:1, v/v/v) at 1.0 mL min^−1^. Retention time (R_t_) for aflatoxins G1, B1, G2, and B2 corresponded to 9, 13, 20, and 32 min for aflatoxins, respectively. Analytical curves were plotted from five different concentrations of standards, from 3.6 to 50 µg kg^−1^ for each aflatoxin. The R^2^ values were higher than 0.99 for all aflatoxins. Recovery experiments were done by spiking the corn grit samples with each aflatoxin solution at levels of 5, 25, and 50 µg kg^−1^ in three replicates. The results showed the recovery values for all aflatoxins in the range of 83.8 ± 9.8% to 105.1 ± 10.6 % for all fortification levels. Limits of Detection (LOD) and Quantification (LOQ) corresponded to 0.8 and 3.6 µg kg^−1^, respectively, for each aflatoxin.

### 2.6. Microbiological Analysis

The enumeration of total fungi was performed in duplicate, according to the official procedures of the Brazilian Ministry of Agriculture, Livestock and Food Supply of Brazil (MAPA) [22]. The 25 g samples were transferred to 500 mL Erlenmeyer flasks, and then a 225 mL portion of 0.1% peptone salt solution was added. Samples were then homogenized for 60 s and the dilutions of 10^−2^, 10^−3^, 10^−4^, and 10^−5^ were obtained using tubes containing 9 mL of 0.1% peptone salt solution. Inocula with 0.1 mL were surface plated on DRBC agar and incubated without reversing at 25 °C for five days in a Bio-Oxygen Demand (BOD) incubator (Tecnal, Piracicaba, São Paulo, Brazil). After this period, total colonies were counted and the results were expressed in CFU g^−1^ of corn grits. The total mesophilic count was conducted in accordance with MAPA [22], as follows. The 25 g samples were transferred to 500 mL Erlenmeyer flasks, and then a 225 mL portion of 0.1% peptone salt solution was added and homogenized. Dilutions from 10^−1^ to 10^−6^ were plated on PCA agar (1 mL inoculum) and then incubated at 36 °C for 48 h. After this period, total colonies were counted and the results were expressed in CFU g^−1^ of corn grits.

### 2.7. Moisture Content and Aw

The moisture content was determined according to the American Association of Cereal Chemists (AACC) [23] using 20 g of ground sample at 130 °C for 1 h. Water activity (A_w_) was verified using an Aqualab CX-2T (Decagon Devices Inc., Pullman, WA, USA) according to the manufacturer’s instructions.

### 2.8. Statistical Analysis

The evaluation of the results was performed by response surface methodology, using STATISCA^®^ 7.0 (software Statistica, Statsoft 7.0, Tulsa, OK, USA). To verify significant difference between the three ozonation conditions applied, ANOVA followed by the Tukey test was performed, using the Sisvar^®^ 5.0 software (UFLA, Lavras, Minas Gerais, Brazil). Descriptive statistics were also calculated using this software.

## 3. Results

### 3.1. Efficacy of Ozonation on Aflatoxin Levels

**Figure 2:**
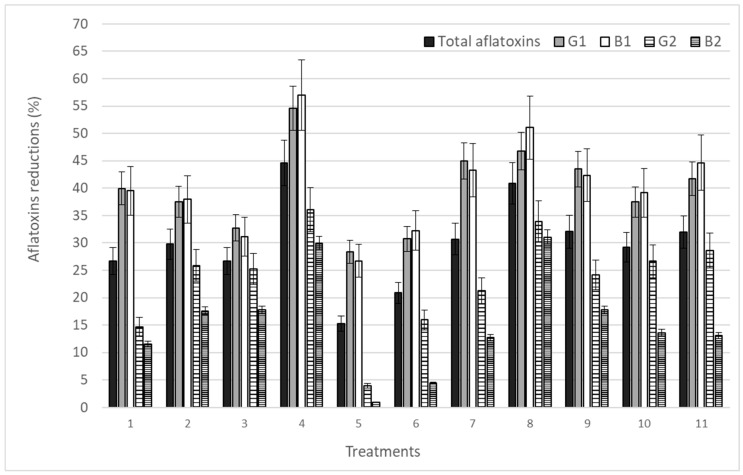
Reduction (%) of total aflatoxin. G_1_, B_1_, G_2_, and B_2_ levels in corn grits after O_3_ exposure under different conditions.

In the present study, using 11 treatments applied to corn grits, it was possible to obtain different reduction percentages in the levels of the four aflatoxins studied (Figure 2). The greatest reductions for AFG1, AFB1, AFG2, and AFB2 corresponded to 54.6%, 57.0%, 36.1%, and 30.0%, respectively, and were obtained in treatment 4, corresponding to 60 mg/L of O_3_, 480 min exposure time and 1 kg of corn grits. Significant reductions of aflatoxins were also obtained in treatments 8 and 9. AFB2 was the less influenced by ozonation in all treatments.

**Figure 3:**
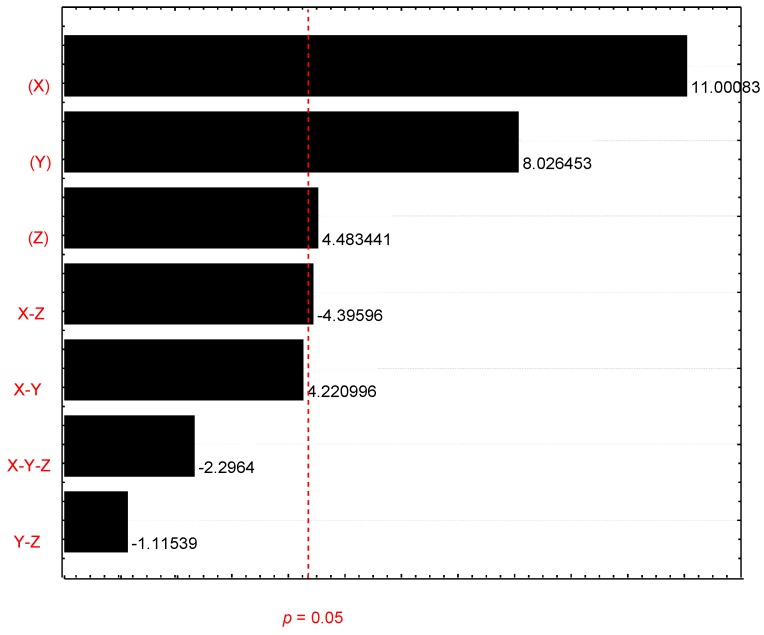
Pareto chart for variables. Total aflatoxin reductions (%) after O_3_ exposure.

Because of the full 2^3^ factorial design, it was possible to study the effects of each independent variable used in the study and its interactions on aflatoxin levels after ozone treatment. The major influences on aflatoxin levels were O_3_ concentration and exposure time. Thus, both variables exerted a positive influence on the reduction of total aflatoxins. As shown in the Pareto chart (Figure 3), the variables X (ozone concentration), Y (time), Z (grain mass), and X-Z interactions were significant in reducing total aflatoxin levels under the conditions studied. The interaction X-Y presented a *p*-value corresponding to 0.0501 and was considered a marginally significant term, not being eliminated in the construction of the mathematical model.

The moisture content percentile of the samples did not differ (*p* > 0.05) between treated and control samples, varying from 8.6 to 9.5. Aw also did not differ, with the overall mean corresponding to 0.53.

### 3.2. Efficacy of Ozonation on Microbiological Count

After the ozonation treatments, the samples with conidia additions of *Aspergillus* spp. and *Fusarium* spp. were evaluated. For the control samples, where only O_2_ was passed through the grits, high counts in both samples were obtained, approximately 1.0 × 10^5^ conidia g^−1^, as expected. Using the highest O_3_ concentration (Treatment 4), it was possible to obtain reductions of 2.04 and 2.77 cycles log CFU g^−1^ of corn grits for the samples plus *Aspergillus* spp. and *Fusarium* spp., respectively. Table 2 presents these results concerning the gaseous ozonation effects on the total fungal counts in the 11 treatments, showing that this process has a fungicidal effect in corn grits. In addition, it is important to highlight the fact that the high numbers of fungal conidia g^−1^ used in the present study are not realistic for this kind of product. We only used these levels to understand the potential of O_3_ in reducing high fungal contamination.

The total mesophilic count was also performed in the ozonized samples without artificial contamination. Some treatments showed negative results (without bacteria growth). For the other treatments, the ozonation reduced the number of CFU g^−1^ to below the limit of determination of the method (1 × 10^1^ CFU g^−1^). The control sample had a count corresponding to 3.4 × 10^3^ CFU g^−1^, whereas in treatment 5, which used the lowest O_3_ concentration and time exposure, the original count was reduced to 7.0 × 10^2^ CFU g^−1^ in corn grits.

## 4. Discussion

Corn contamination by aflatoxins is a global food safety issue [24]. Some studies using ozonation, applied to different kinds of food, have been carried out to evaluate the efficacy of this technology on the degradation of aflatoxins.

As expected, the forms AFG1 and AFB1 were the most influenced by the process due to the presence of double bonds in their molecules (C8–C9). These are first attacked by O_3_, leading to their breakdown into products with lower molecular weight, such as organic acids, aldehydes, and ketones [25,26]. In other studies, higher reductions of aflatoxins by O_3_ on corn kernels have been demonstrated [27,28]. This can be explained by two factors: first, because the surface area of corn grits is higher than for kernels, a higher O_3_ concentration is required; and second, because the moisture content of the grits used in the present study was very low (9.5 ± 0.4%) [28,29].

The higher the moisture content, the greater the potential of mycotoxin degradation, due to the formation of ˙OH radicals from a strong oxidation capacity, as demonstrated by [29]. This information is also validated for mycobiota, since ozonized maize with a high moisture content presented as the most effective in controlling *Aspergillus* spp. and *Fusarium* spp. [30]. In our work, we preferred to use a low moisture content since it is the condition under which corn grits are stored in the food industry, and because this raw material has not yet been studied with respect to ozonation. Even so, the results found here demonstrate that gaseous ozonation can be a good method to remediate corn grit contamination by aflatoxins, especially regarding AFB1 and AFG1 contamination. Other studies on corn dealing with the detoxification of the mycotoxins zearalenone and ochratoxin [31] and the degradation of pirimiphos-methyl residues [32] show a great reduction of those compounds after ozone application. Those results are in accordance with our study, indicating that ozone exhibits a remarkable potential in reducing various mycotoxins and toxic compounds in corn while minimally affecting its quality.

Similar results for the reduction of fungal contamination in wheat samples by ozonation were previously reported [16], with values around 3.0 cycles log CFU g-1 reductions in total fungal count. This reduction of microbial cells by O_3_ can be related to cell metabolism alterations, leading to apoptosis and oxidative stress, which makes the use of this technology very interesting for controlling toxigenic fungal and bacterial development, which are among the main food contamination problems [33]. Additionally, the use of ozone to control microbial contamination, especially in grains, has an advantage over other chemical agents because it is considered environmentally friendly to produce and its use does not leave any residues in the food, since the O_3_ dissociates into oxygen. For this reason, ozonation is classified as a green technology [34].

## 5. Conclusions

Gaseous ozonation is an effective non-thermal technology to reduce aflatoxins and microbial contamination in corn grits. Using different concentrations of O_3_ by full factorial design, it was possible to obtain reductions of 54.6%, 57.0%, 36.1%, and 30.0% for AFG1, AFB1, AFG2, and AFB2, respectively. Fungal contamination was reduced around 3 cycles log CFU g^−1^. The same microbicidal effect was verified in the total mesophilic count. Since corn grits are a raw material used to produce a large variety of foods, gaseous ozonation can be considered a very promising option to improve their safety, by effectively reducing aflatoxin and microbial contamination.

## Figures and Tables

**Table 1:** Matrix of the full factorial design with coded and real values.

Treatments	O_3_ Concentration (mg/L)	Exposure Time (min)	Mass of grains (kg)
1	−1 (20)	−1 (120)	−1 (1)
2	−1 (20)	+1 (480)	−1 (1)
3	+1 (60)	−1 (120)	−1 (1)
4	+1 (60)	+1 (480)	−1 (1)
5	−1 (20)	−1 (120)	+1 (5)
6	−1 (20)	+1 (480)	+1 (5)
7	+1 (60)	−1 (120)	+1 (5)
8	+1 (60)	+1 (480)	+1 (5)
9	0 (40)	0 (300)	0 (3)
10	0 (40)	0 (300)	0 (3)
11	0 (40)	0 (300)	0 (3)

**Table 2:** Total fungal and mesophilic bacteria counts in the corn grit samples after exposure to different O_3_ conditions. C^1^—Control samples. * LQ = 1 X 10^1^ UFC g^−1^

Treatments	Samples Spiked with *Fusarium* spp. Conidia		Samples Spiked with *Aspergillus* spp. Conidia		Total Mesophilic Count * (CFU/g)
	Count UFC/g	Results log10 N_0_/N	Count UFC/g	Results log10 N_0_/N	
**1**	5.0 × 10^4^	1.10	5.4 × 10^4^	1.08	4.0 × 10^2^
**2**	5.5 × 10^3^	1.34	6.5 × 10^3^	1.33	<LQ
**3**	1.2 × 10^3^	1.64	1.5 × 10^3^	1.62	<LQ
**4**	6.5 × 10^1^	2.77	3.1 × 10^2^	2.04	<LQ
**5**	4.6 × 10^4^	1.08	3.0 × 10^4^	1.14	7.0 × 10^1^
**6**	3.5 × 10^4^	1.11	1.6 × 10^4^	1.21	1.6 × 10^2^
**7**	4.2 × 10^3^	1.39	3.4 × 10^4^	1.12	1.1 × 10^2^
**8**	5.9 × 10^3^	1.33	2.6 × 10^3^	1.49	<LQ
**9**	3.9 × 10^4^	1.11	1.5 × 10^4^	1.22	1.4 × 10^2^
**10**	4.3 × 10^4^	1.09	1.5 × 10^4^	1.21	1.3 × 10^2^
**11**	6.3 × 10^4^	1.05	6.2 × 10^4^	1.08	1.2 × 10^2^
**C^1^**	1.1 × 10^5^	1.00	1.2 × 10^5^	1.00	3.4 × 10^3^
